# Insights into the regulation of molecular mechanisms involved in energy shortage in detached citrus fruit

**DOI:** 10.1038/s41598-019-57012-7

**Published:** 2020-01-24

**Authors:** Paco Romero, Fernando Alférez, Beatriz Establés-Ortiz, María T. Lafuente

**Affiliations:** 1 Department of Food Biotechnology, Instituto de Agroquímica y Tecnología de Alimentos, IATA-CSIC, Av. Agustín Escardino 7, 46980 Paterna Valencia, Spain; 2 Present Address: Southwest Florida Research and Education Center, University of Florida-IFAS, Immokalee, FL 34142 USA

**Keywords:** Plant molecular biology, Abiotic

## Abstract

Harvested fruit undergo carbon and energy deprivation. However, the events underlying this energy-related stress in detached fruit and their involvement in cell damage have not yet been elucidated. We showed that supplementing detached sweet oranges with additional carbon or energy sources reduced peel damage, while inhibitors of energy metabolism increased it. We investigated the effect of an exogenous source of carbon (glycerol), energy (ATP), and an inhibitor of energy metabolism 2-deoxy-D-glucose (DeOGlc) + sodium iodoacetate (IAc), on the transcriptome of harvested fruit flavedo (outer peel part). ATP and Gly induced common, but also specific, alternative modes of energy metabolism by reducing the stress caused by energy shortage. They also induced shifts in energy metabolism that led to the production of the intermediates required for plant defense secondary metabolites to form. ATP and Gly triggered changes in the expression of the genes involved in cell lesion containment through a defined pathway involving hormones and redox-mediated signaling. DeOGlc + IAc had a contrasting effect on some of these mechanisms. These chemicals altered the biological processes related to membrane integrity and molecular mechanisms involving reactive oxygen species (ROS) production, and lipid and protein degradation.

## Introduction

Harvested fruit undergo nutrient deprivation, an important abiotic stress due to fruit detachment from plants. However, knowledge about the basic mechanisms underlying energy shortage caused by starvation in harvested fruit and their putative involvement in cell damage and postharvest losses is scarce^1,2^. Metabolic responses to starvation in nonphotosynthetic tubers containing high carbon respiratory sources have been studied^3,4^. Detached nonphotosynthetic sink organs, like citrus fruit peel, have limited carbon sources, but very little is known about the adaptive mechanisms required to balance carbon and energy demand in these tissues. Previous research by our group revealed that the application of 1-methylcyclopropene (1-MCP) to detached citrus fruit markedly increased its respiration, which is indicative of a higher energy demand. 1-MCP led to membrane and cellular damage, and also to a peel damage disorder called nonchilling peel pitting (NCPP). This disorder is manifested as depressed damaged areas on peel^5,6^ (Supplementary Fig. S1). This result indicates a link between starvation and peel damage, although 1-MCP also has some toxic effects on fruit^6^. Further research has shown that lowering storage temperature by 10 °C markedly delays NCPP developing in sweet oranges^7^. Lowering the storage temperature may delay the metabolic changes that lead to NCPP symptom development. However, a drop of 10 °C suppresses the respiration rate by ~2-fold in detached fruit and vegetables^8^, which involves a drastic reduction in the carbon/energy demand needed to sustain respiration. Therefore, a putative link between reduced fruit energy demand in detached fruit and diminished peel damage cannot be ruled out. By considering that: (1) fruit detachment comes at an energy cost because fruit respiration must be sustained in the absence of nutrient translocation from trees; (2) the cellular perception of energy shortage i.e. a drop in adenosine triphosphate (ATP), may affect membrane integrity;^9,10^ our working hypothesis was that fruit detachment may regulate complex mechanisms associated with carbohydrate starvation and energy metabolism, which might lead to peel damage in citrus fruit. To test this hypothesis, we used NCPP development as an indicator of damage caused by energy stress. As detached fruit have to use their own respiratory substrates to maintain respiration, we examined the effect of (1) external energy sources, which may be used as a substrate for respiration, or for preventing autophagy^11,12^ and (2) inhibitors that interfere with energy metabolism^10^ on the susceptibility of citrus fruit to develop peel damage. Based on these results, we performed a comparative transcriptomic analysis between selected treatments to unravel the molecular mechanisms underlying energy stress that led to peel damage in citrus fruit. These chemicals could alter the consumption of fruit natural sources of energy, but may also have additional unintended effects. Therefore, we further examined the ATP-induced changes on the transcriptome and their effect on tissue damage.

## Results

### Citrus fruit peel damage development depends on energy status

The effect of different external energy sources and inhibitors of energy metabolism on NCPP, which may favor lipid hydrolysis, was investigated. Based on the successful results obtained in previous research into plant cells^10–12^, sucrose (Suc) and glycerol (Gly) were selected as additional energy sources. The combination of azide (AZ) + salicylhydroxamic acid (SHAM), and that of 2-deoxy-D-glucose (DeOGlc) + sodium iodoacetate (IAc), were used to disrupt energy metabolism. Gly may favor the supply of respiratory substrates to mitochondria via ATP production;^11,12^ and Suc can be easily broken down to hexoses in citrus fruit peel^13^. AZ inhibits at the end of the cytochrome pathway, whereas SHAM blocks the alternative oxidase pathway and DeOGlc + IAc the glycolytic pathway^10^. In this work, NCPP was used as an indicator of energy shortage-induced damage. As NCPP symptoms may also develop under water^14,15^ or atmospheric^16^ stress, all the experiments were performed in sweet oranges stored under conditions that minimized environmental stress (90–95% relative humidity (RH) and 20 °C) and did not alter the storage atmosphere (unwaxed fruit kept in open plastic boxes). Whether Gly and Suc were able to reduce NCPP was first tested in mature Navelina (*Citrus sinensis* (L.) Osbeck) sweet oranges. NCPP occurred after 3 days (Table 1). By day 5, NCPP was very low (0.40 on a rating scale from 0 to 4) in the control fruit, no NCPP damage was evident in the Gly-treated fruit, and the Suc-treated fruit presented less damage compared to the control fruit. The effect of Suc was lost by day 9, but the control fruit still showed more NCPP damage than the Gly-treated fruit (Table 1). We confirmed the efficacy of Suc and Gly in reducing NCPP in Navelate (*Citrus sinensis* (L.) Osbeck) sweet oranges (Table 1). The DeOGlc + IAc combination was the most effective in interfering with energy metabolism by accelerating NCPP damage development (Table 1).

**Table 1:** The NCPP index of the Navelina and Navelate oranges treated for 2 min with Suc and Gly 10 mM, 50 mM AZ + 50 mM SHAM, 50 mM DeOGlc + 5 mM IAc and stored in the dark at 20 °C and 90–95% RH. Values are the means of three biological replicates. ^a,b,c,d^Different letters mean significant differences (p ≤ 0.05) between the control fruit and those treated with each specific treatment for the same analysis day.

NCPP index (rating scale 0–4)						
Treatment	Navelina			Navelate		
	3 days	5 days	9 days	3 days	5 days	9 days
Control	0.00	0.40^a^	0.96^a^	0.10^b,c^	0.67^b^	0.67^c^
Sucrose	0.00	0.24^b^	0.91^a^	0.05^c^	0.20^c^	0.52^c,d^
Glycerol	0.00	0.00^c^	0.58^b^	0.00^c^	0.30^c^	0.42^d^
AZ + SHAM	nd	nd	nd	0.30^b^	0.63^b^	1.11^b^
DeOGlc + IAc	nd	nd	nd	1.34^a^	2.38^a^	2.38^a^

nd = non-determined.

### Effect of Gly and Suc on ATP levels in citrus fruit peel and of exogenous ATP on NCPP

**Figure 1:**
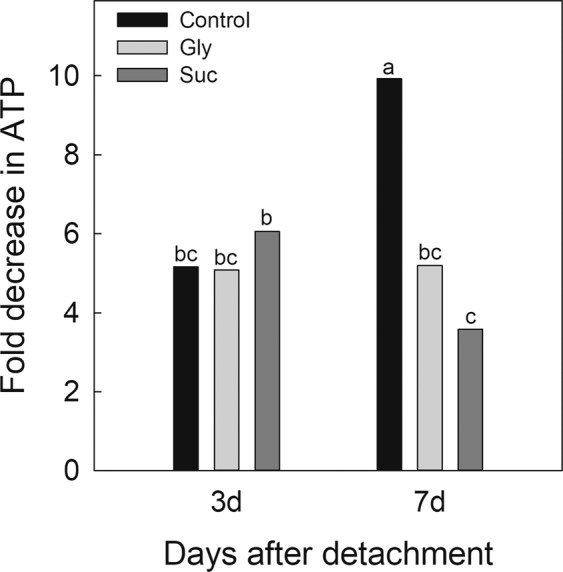
Effect of sucrose and glycerol on ATP content in the flavedo of mature Navelate oranges after fruit detachment. ATP levels lowered in all the samples. Thus the drop in ATP was determined and expressed as a fold-decrease vs. the ATP levels in the flavedo of the freshly harvested fruits. Fruit were kept in the dark at 20 °C and 90–95% RH. Values are the means of three biological replicates. Different letters mean significant differences (p ≤ 0.05) between the control and treated fruit.

**Figure 2:**
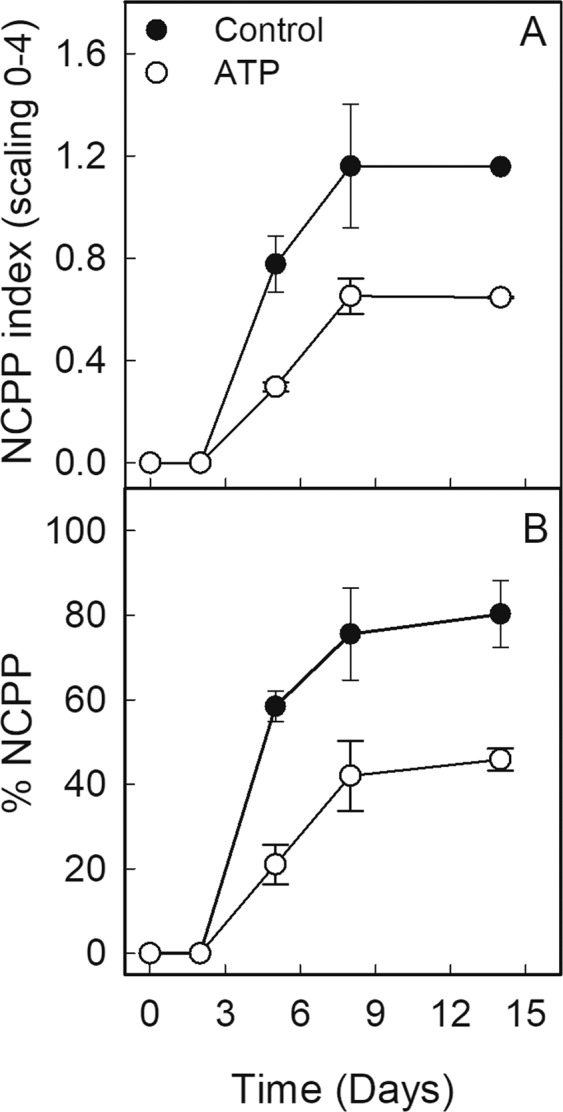
Changes in NCPP incidence, estimated as % of fruit with NCPP, and in NCPP severity (NCPP index) in the fruit treated (white circle), or not (black circle), with ATP (5 mM). A rating scale from 0 to 4 was used to estimate NCPP severity (Supplementary Fig. S1). ATP was applied to the freshly harvested fruit and every 3 days thereafter. Values are the means of three replicates of 10 fruit each ± SEM. Significant differences (p ≤ 0.05) among the control fruit and those treated with ATP were found after 2 days when comparing the same analysis day.

ATP content lowered in the outer peel part (flavedo) of the control Navelate fruit during storage, but this reduction was ~2-fold lower by day 7, when Suc or Gly were applied (Fig. 1). In view of this result, we examined the effect of treating fruit with ATP on NCPP development, and found that it reduced both damage severity and incidence (Fig. 2).

### Changes induced by Gly and DeOGlc + IAc in the transcriptome of flavedo of citrus fruit

Since DeOGlc + IAc had a stronger effect than AZ + SHAM on increasing NCPP, it was selected as an inhibitor of energy metabolism to examine changes in the transcriptome. Gly, rather than Suc, was selected for the transcriptomic analysis as Suc is a signaling molecule in plant stress responses^17^. Gly decreased the reduction in ATP by day 7 (Fig. 2). Therefore, the flavedo samples from the Gly-treated fruit and their respective controls (untreated fruit) were taken by day 7. The DeOGlc + IAc-treated sample and its control were taken by day 3 because of the considerable damage that occurred after this time point when this inhibitor was applied (Table 1).

Gly and DeOGlc + IAc regulated many differentially expressed genes (DEGs). The number of DEGs showing at least a 2-fold change in expression (SAM, FDR < 0.01) is summarized in Supplementary Fig. S2A. A large number of DEGs was found in the DeOGlc + IAc-treated fruit. The repeatability of the microarray data across replications was validated by a Principal Component Analysis (PCA) (Supplementary Fig. S2B), and by Hierarchical Cluster (HCA) and Heatmap analyses (Supplementary Fig. S2C). The PCA revealed marked differences in the gene expression patterns among the freshly harvested (FH) fruit and those treated with Gly or DeOGlc + IAc. Accordingly, replicates of the same conditions were grouped together in the HCA (Supplementary Fig. S2C).

### Functional analysis based on transcriptome profiles

**Figure 3:**
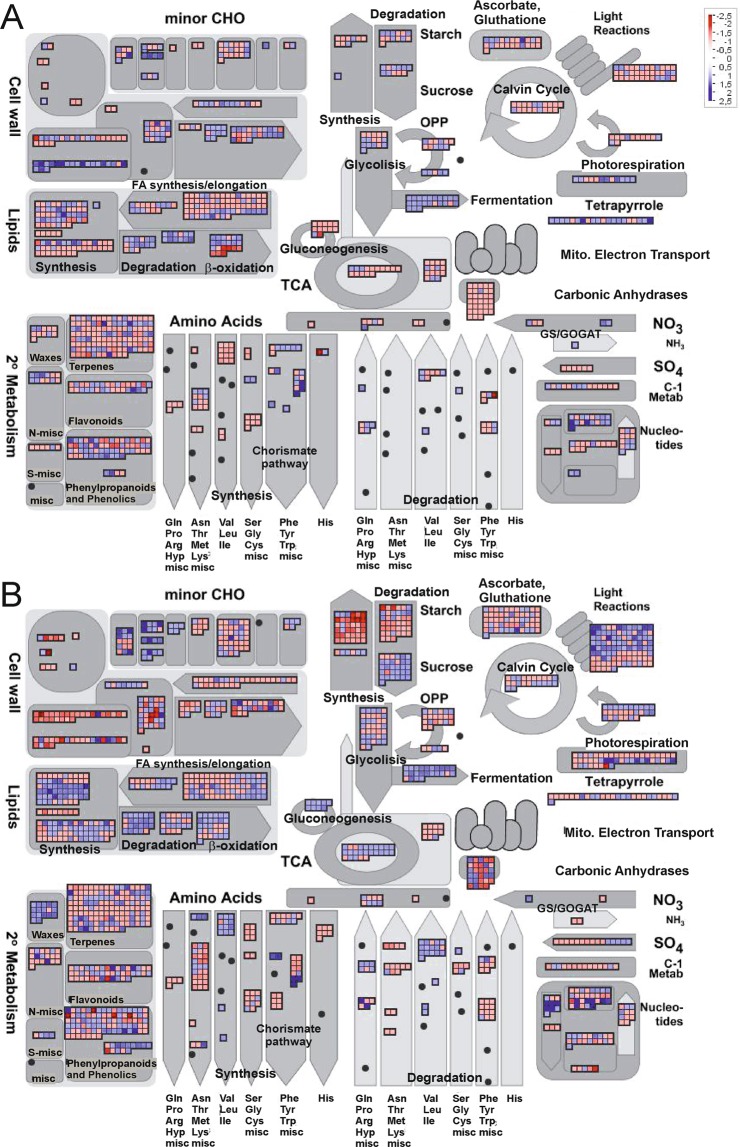
Metabolic overview using MapMan^36^ to compare transcript accumulation in the flavedo of the Gly- (**A**) and ATP-treated (**B**) fruit vs. their untreated control samples. Fruit were kept in the dark at 20 °C and 90–95% RH. Red and blue squares represent the genes with decreasing and increasing transcript levels in the treated fruit, respectively, vs. the control samples. The color scale is indicated in the figure.

**Figure 4:**
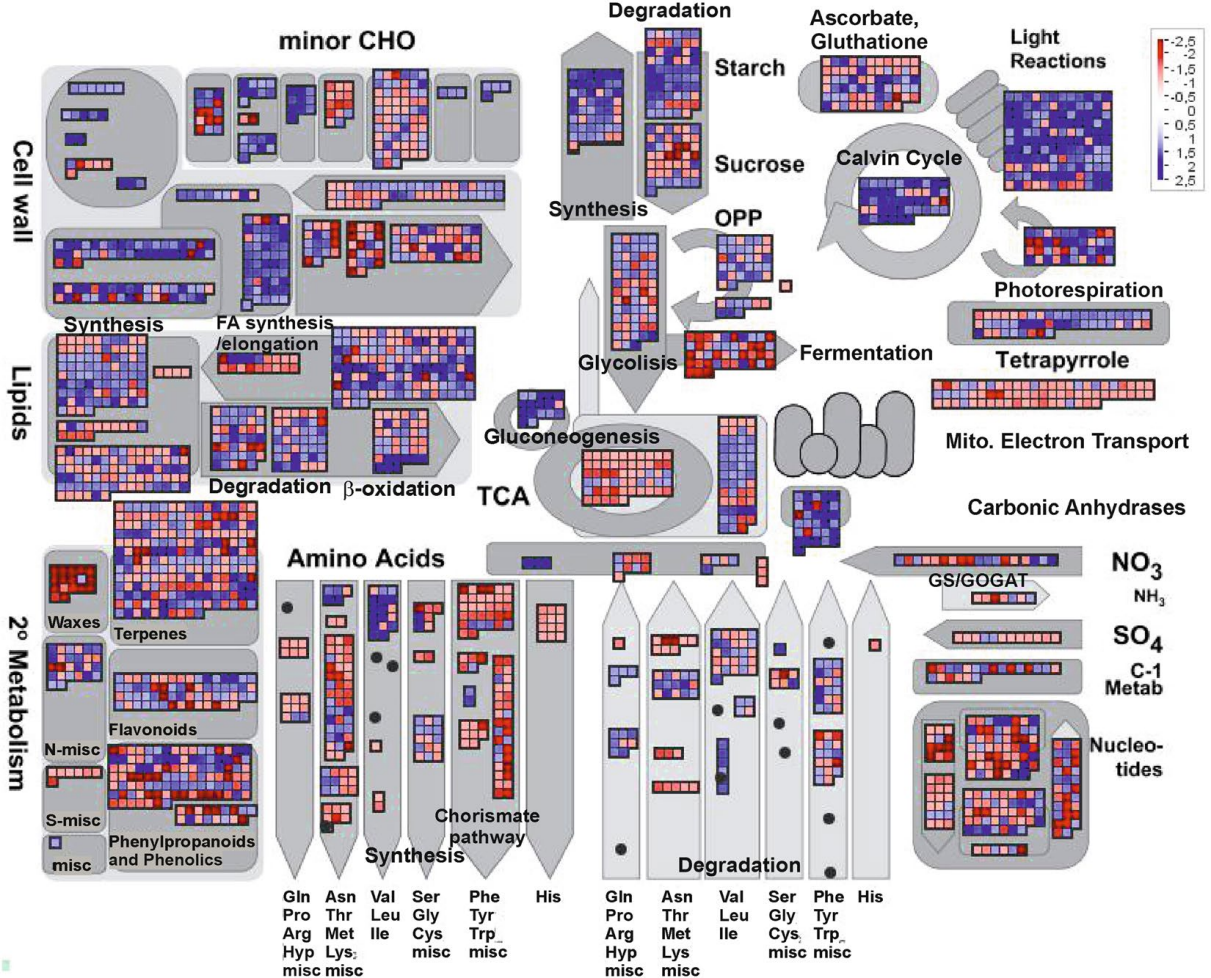
Metabolic overview using MapMan^36^ when comparing transcript accumulation in the flavedo of DeOGlc + IAc-treated fruit vs. their untreated control samples. Fruit was kept in the dark at 20 °C and 90–95% RH. Red and blue squares represent the genes with decreasing and increasing transcript levels in the treated fruit, respectively, vs. the control samples. The color scale is indicated in the figure.

**Figure 5:**
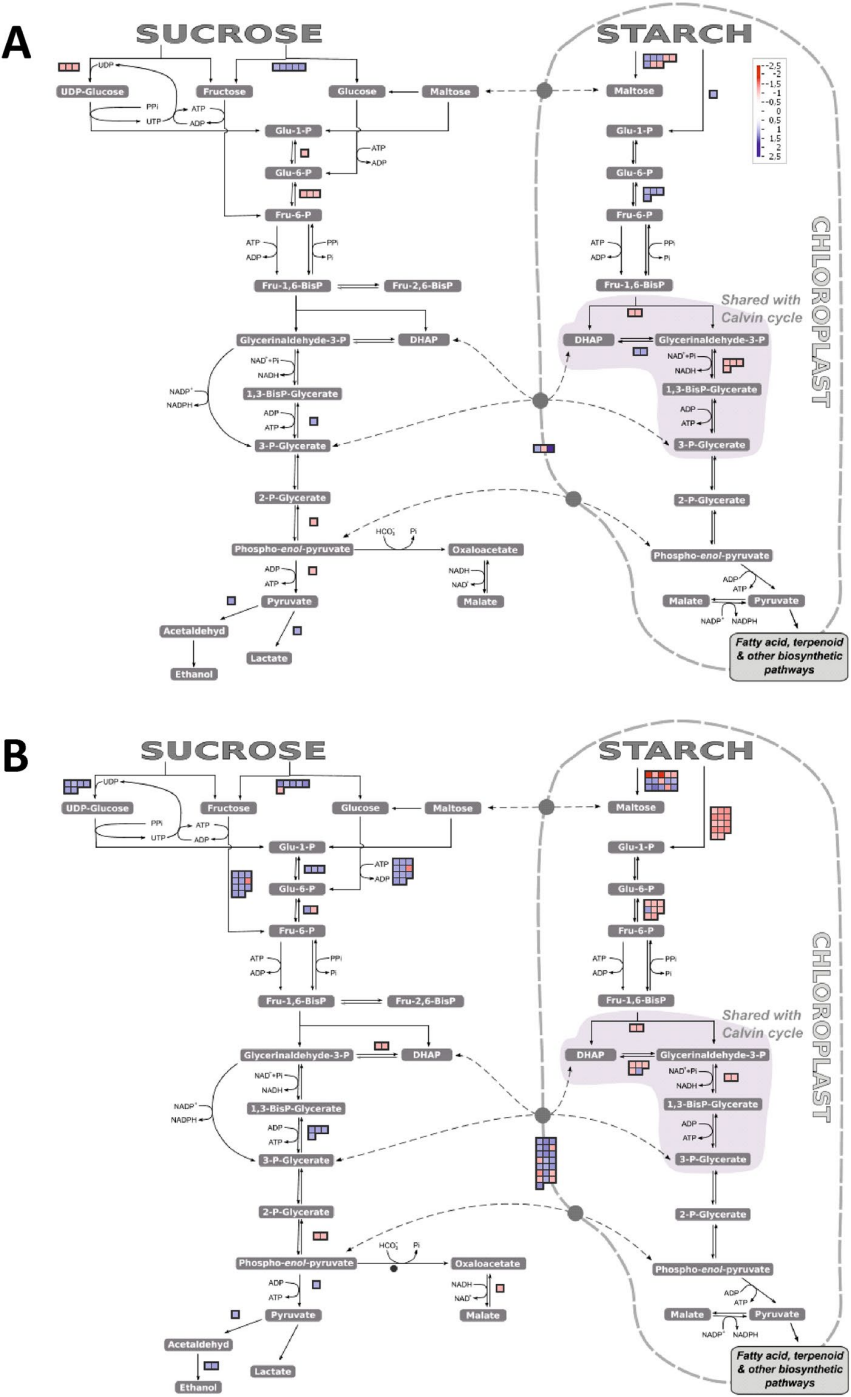
The glycolysis pathway detail using MapMan^36^ when comparing transcript accumulation in the flavedo of the Gly- (**A**) and ATP-treated (**B**) fruit vs. their untreated control samples. Fruit were kept in the dark at 20 °C and 90–95% RH. Red and blue squares represent the genes with decreasing and increasing transcript levels in the treated fruit, respectively, vs. the control samples. The color scale is indicated in the figure.

**Figure 6:**
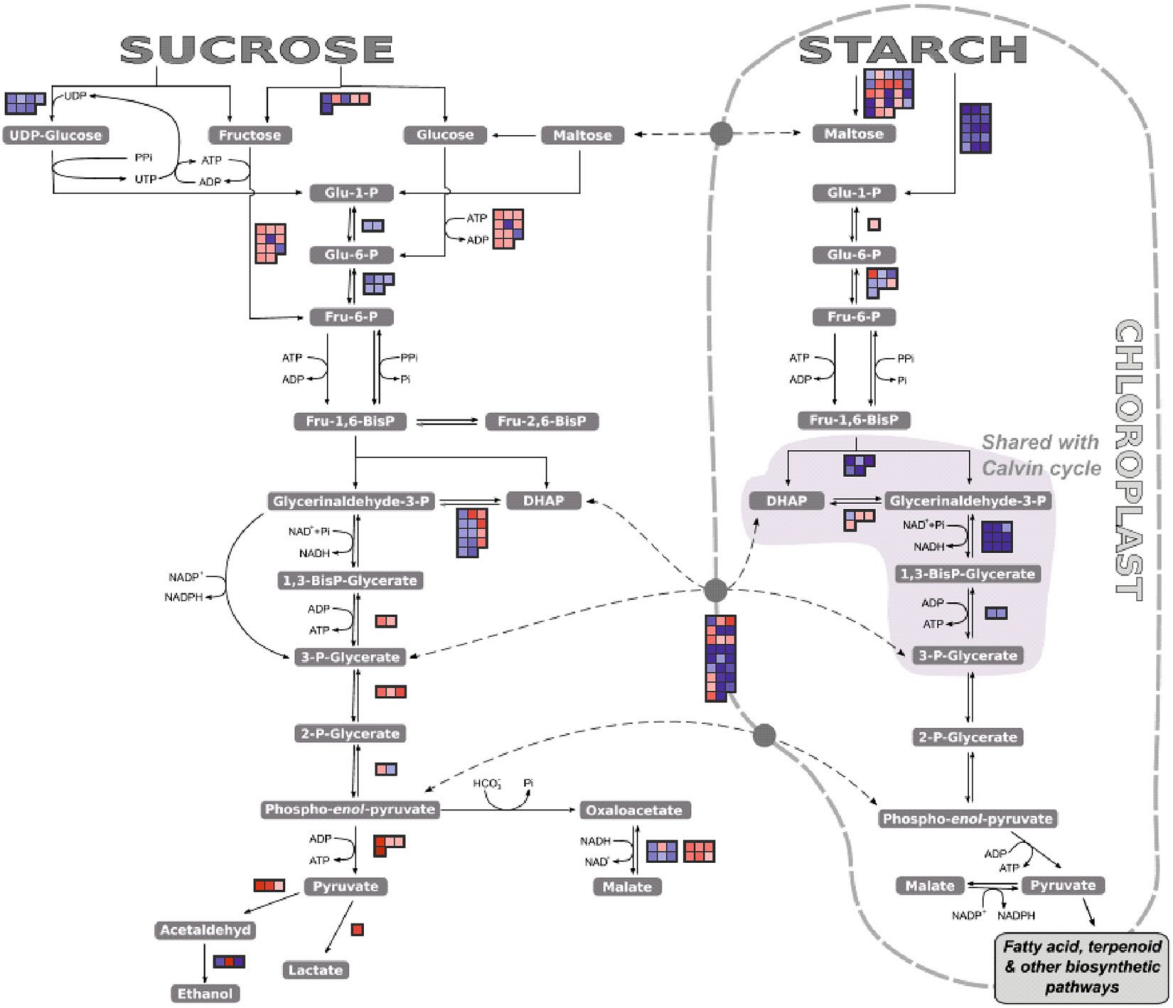
The glycolysis pathway detail using MapMan^36^ when comparing transcript accumulation in the flavedo of the DeOGlc + IAc-treated fruit vs. their untreated control samples. Fruit were kept in the dark at 20 °C and 90–95% RH. Red and blue squares represent the genes with decreasing and increasing transcript levels in the treated fruit, respectively, vs. the control samples. The color scale is indicated in Fig. 5.

The gene ontology (GO) analysis revealed the key processes represented in the sets of DEGs in the Gly- and DeOGlc + IAc-treated fruit. Biological processes (BP) (Table 2), and cellular components (CC) and molecular functions (MF) (Supplementary Table S1), were examined. The differential processes regulated by both treatments were also compared with those altered by ATP because, like Gly, ATP may provide energy and reduce NCPP (Table 1; Fig. 2). The ATP treatment induced 135 and repressed 56 DEGs, with at least a 2-fold change in gene expression. The effect of ATP on the transcriptome of flavedo was examined on day 3, when differences in NCPP began to become evident. The differential BP were grouped according to their regulation patterns by considering the putative ability of treatments to provide carbon/energy and to reduce NCPP (Gly and ATP), or to inhibit energy metabolism and to increase the disorder (DeOGlc + IAc). Pattern 1 grouped the BP over-represented in both the Gly- and ATP-treated samples and infra-represented in the DeOGlc + IAc-treated fruit (Table 2). These processes could be, therefore, the best candidates to be involved in citrus fruit resistance to develop damage caused by energy shortage. They were related mainly to the biosynthesis of secondary metabolites, including stilbene, coumarin, tyrosine and lignin. The BP in pattern 2 were repressed by DeOGlc + IAc, were induced only by Gly, and were related to salicylic acid (SA) metabolism, senescence and the response to nitrogen. Moreover, all the MF displayed esterase activity when acting on different hormones (Supplementary Table S1). Pattern 3 in Table 2 grouped the processes that were contrarily regulated by DeOGlc + IAc and ATP. The oxidoreduction-related BP and MF, and BP participating in the perception of hormone ethylene and in the chorismate and the subsequent phenylpropanoid metabolic pathways were induced by ATP and repressed by DeOGlc + IAc. The BP and MF infra-represented by ATP in this pattern were involved in energy, carbohydrate, starch and Suc metabolism, and also in the degradation of complex carbohydrates of cell walls (Table 2, Supplementary Table S1). Pattern 4 included the processes commonly over-represented by Gly and ATP, which were not regulated by DeOGlc + IAc. They participated in the response of plants to stress (Table 2). Some processes were regulated only by Gly (pattern 5) and participated in cell wall loosening, regulation of transcription, and isoprenoid and ketone metabolism. Pattern 6 grouped the processes that were regulated only by ATP. The over-represented BP were related to both phosphorylation and the defense of plants against reactive oxygen species (ROS) and other stresses, or were involved in the pyrimidine nucleobase metabolic process (Table 2). These over-representations agreed with the MF and CC induced by ATP (Supplementary Table S1). In contrast, the ATP infra-represented BP were mainly related to Suc metabolism and to cell wall biogenesis and catabolism, whereas the MF performed diverse functions. The number of processes specifically induced and repressed by DeOGlc + IAc was abundant (Supplementary Table S2). They reflected the strong impact of the inhibitor to cause oxidative stress and to alter energy and carbohydrate metabolism. The infra-represented BP were related to the shikimate and the chorismate biosynthetic processes, and also to the subsequent phenylpropanoids and other secondary metabolites. DeOGlc + IAc also altered diverse CC that involved plastids, the apoplast, amyloplast, membranes, glyoxysome, as well as stromules and the glycolate oxidase complexes (Supplementary Table S2). The visualization of how the transcriptomic changes were distributed into the general overview and concrete metabolic pathways (MapMan analysis; Figs. 3–6 and Supplementary Figs. S3 and S4) agreed with the GO analyses (GOStats analysis; Table 2 and Supplementary Tables S1 and S2). This indicates that both Gly and ATP induced common and/or exclusive mechanisms in order to cope with the energy stress caused by carbon deprivation, but also plant defense responses (Figs. 3A,B, 5A,B). These processes were generally counteracted by DeOGlc + IAc (Figs. 4 and 6, and Supplementary Figs. S3C and S4C). The three treatments had a different effect on the cell wall: ATP and Gly affected Suc and starch metabolism and the TCA cycle, and up-regulated the fermentation- and nucleotide-related genes (Fig. 3A,B). Only Gly favored tetrapyrrole metabolism, inhibited glucogenesis (Fig. 3A,B) and down-regulated the β-oxidation-related genes. DeOGlc + IAc had a marked effect on both the synthesis and degradation of lipids (Fig. 4), and over-represented the glyoxysome CC (Supplementary Table S2). It also up-regulated abundant gluconeogenesis-related genes, and had a clear effect on the regulatory mechanisms involving the modification and degradation of proteins (Supplementary Fig. S3C), and also on important steps of glycolysis (Fig. 6) and the TCA cycle (Supplementary Fig. S4C). DeOGlc + IAc led to the infra-representation of SA and jasmonic acid (JA), which were over-represented by ATP (Supplementary Fig. S3B). Gly had a marked effect on JA (Supplementary Fig. S3A). Lastly, MapMan envisaged that the three treatments would induce ROS detoxification-related responses (Supplementary Fig. S3), which could vary with treatment, and that the major changes related to ROS were induced by DeOGlc + IAc.

**Table 2:** Gene ontology (GO) analysis (GOStats, P < 0.05) of the biological processes (BP) over- (↑) or infra-represented (↓) in the flavedo of the Gly-, DeOGlc + IAc- and ATP-treated fruit vs. their untreated control samples.

GO Code	GO Term	Gly	ATP	DeOGlc + IAc
**Pattern 1: Commonly regulated by Gly and ATP, and contrary to DeOGlc + IAc**				
GO:0006570	tyrosine metabolic process	↑	↑	↓
GO:0009805	coumarin biosynthetic process	↑	↑	↓
GO:0009809	lignin biosynthetic process	↑	↑	↓
GO:0009811	stilbene biosynthetic process	↑	↑	↓
**Pattern 2: Contrarily regulated by Gly and DeOGlc + IAc**				
GO:0009696	salicylic acid metabolic process	↑		↓
GO:0010150	leaf senescence	↑		↓
GO:0010243	response to organic nitrogen	↑		↓
**Pattern 3: Contrarily regulated by ATP and DeOGlc + IAc**				
GO:0000162	tryptophan biosynthetic process		↑	↓
GO:0009094	L-phenylalanine biosynthetic process		↑	↓
GO:0009727	detection of ethylene stimulus		↑	↓
GO:0009835	fruit ripening		↑	↓
GO:0032259	methylation		↑	↓
GO:0051555	flavonol biosynthetic process		↑	↓
GO:0055114	oxidation-reduction process		↑	↓
GO:0005978	glycogen biosynthetic process		↓	↑
GO:0006011	UDP-glucose metabolic process		↓	↑
GO:0019252	starch biosynthetic process		↓	↑
**Pattern 4: Commonly regulated by Gly and ATP**				
GO:0009269	response to desiccation	↑	↑	
GO:0009651	response to salt stress	↑	↑	
GO:0009741	response to brassinosteroid stimulus	↑	↑	
GO:0010089	xylem development	↑	↑	
GO:0010112	regulation of systemic acquired resistance	↑	↑	
**Pattern 5: Regulated by Gly**				
GO:0006355	regulation of transcription, DNA-dependent	↑		
GO:0008299	isoprenoid biosynthetic process	↑		
GO:0009631	cold acclimation	↑		
GO:0009828	plant-type cell wall loosening	↑		
GO:0019953	sexual reproduction	↑		
GO:0042180	cellular ketone metabolic process	↑		
GO:0019438	aromatic compound biosynthetic process	↓		
GO:1901362	organic cyclic compound biosynthetic process	↓		
GO:1901607	alpha-amino acid biosynthetic process	↓		
**Pattern 6: Regulated by ATP**				
GO:0002213	defense response to insect		↑	
GO:0006206	pyrimidine nucleobase metabolic process		↑	
GO:0006388	tRNA splicing, via endonucleolytic cleavage and ligation		↑	
GO:0006541	glutamine metabolic process		↑	
GO:0006801	superoxide metabolic process		↑	
GO:0007205	protein kinase C-activating G-protein coupled receptor signaling pathway		↑	
GO:0009312	oligosaccharide biosynthetic process		↑	
GO:0009693	ethylene biosynthetic process		↑	
GO:0042432	indole biosynthetic process		↑	
GO:0042542	response to hydrogen peroxide		↑	
GO:0005985	sucrose metabolic process		↓	
GO:0009739	response to gibberellin stimulus		↓	
GO:0009833	primary cell wall biogenesis		↓	
GO:0010214	seed coat development		↓	
GO:0010951	negative regulation of endopeptidase activity		↓	
GO:0016049	cell growth		↓	
GO:0016998	cell wall macromolecule catabolic process		↓	
GO:0043622	cortical microtubule organization		↓	

Processes were grouped according to their regulation pattern by considering the ability of treatments to provide energy or to interfere with energy metabolism. Three biological replicates from each condition were used.

### Effect of Gly, ATP and DeOGlc + IAc on ethylene production

**Figure 7:**
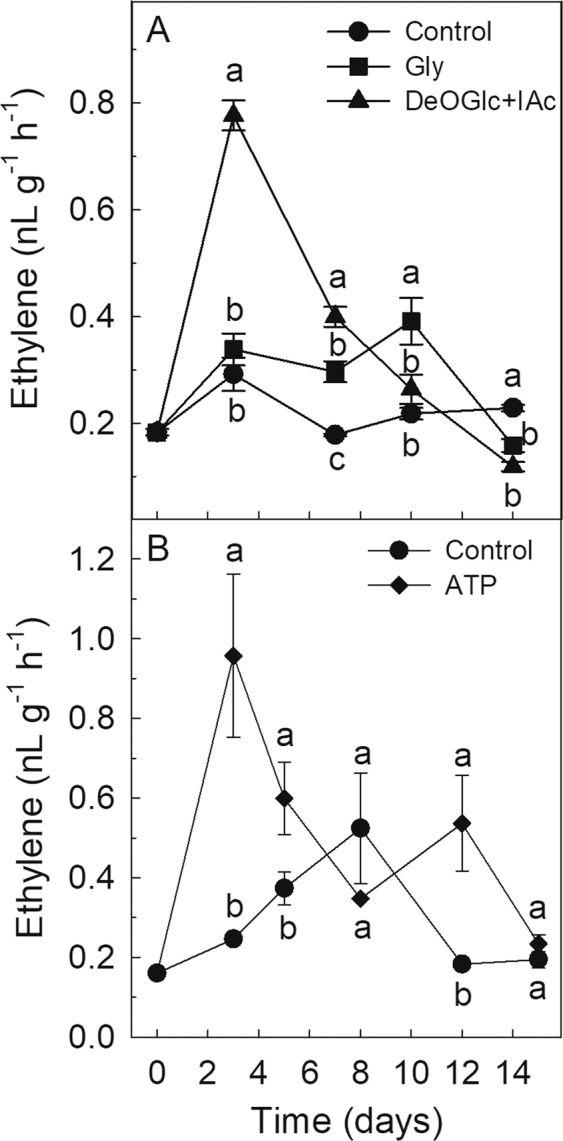
Effect of Gly, DeOGlc + IAc (**A**) and ATP (**B**) on ethylene production of the flavedo of Navelate oranges. Values are means of three replicate samples ± SEM. Different letters mean significant differences (p ≤ 0.05) between the control fruit and those treated with Gly, DeOGlc + IAc or ATP for the same analysis day.

Treating citrus fruit with ethylene reduces NCPP severity^7^. Therefore, the evolution of this hormone was monitored in the flavedo of the detached fruit treated with Gly, ATP and DeOGlc + IAc. DeOGlc + IAc and ATP induced a marked transient increase in ethylene production, which peaked by day 3. The levels of the hormone in the flavedo of the Gly-treated fruit almost doubled those of the control fruit by days 7 and 10 (Fig. 7).

## Discussion

The results showing that Suc, Gly and ATP decreased NCPP in citrus fruit (Table 1 and Fig. 2), and that Gly and Suc reduced the decline in ATP that occurred after detachment (Fig. 1), agree with the idea that the cellular perception of energy shortage may cause cell damage^9,10^. They also reinforce that carbon starvation and energy shortage are important factors for NCPP to develop in the detached citrus fruit kept under non environmental stresses^5,6^.

The GO and MapMan analyses unraveled that ATP and Gly induced common, but also specific, responses that can be grouped in: (1) metabolic shifts for energy conservation to reduce the negative impact of carbon shortage on detached fruit; (2) the generation of secondary metabolites needed to contain cell damage propagation, which may be mediated by hormones and redox signaling. Some responses were contrarily regulated by DeOGlc + IAc, which also induced specific responses.

Among the responses related to energy metabolism, both Gly and ATP increased the expression of the fermentation-related genes (Fig. 3A,B), while Gly only repressed gluconeogenesis and the expression of the genes involved in lipid β-oxidation (Fig. 3A). Fermentation favors energy production and, in starved cells, can prevent lipid degradation^10^, which has been related to NCPP development^6^_._ On the contrary, DeOGlc + IAc repressed the genes involved in fermentation, while favoring gluconeogenesis (Fig. 4), which generates glucose from the breakdown of lipids, proteins or Gly. Moreover, it repressed most genes in the first glycolysis steps (Fig. 6) and the genes encoding phosphoenolpyruvate carboxylase (PEPC) (Fig. 6, Supplementary Table S3), which means favoring the replacement of sugars with lipids or proteins to sustain respiration^18^. This inhibitor negatively affected energy production by down-regulating not only the genes involved in the conversion of pyruvate into Acetyl-CoA, but also those participating in nearly every step in the TCA cycle and the mitochondrial electron transport chain (Supplementary Fig. S4C). Accordingly, DeOGlc + IAc induced the genes involved in the metabolism and β-oxidation of lipids (Fig. 4), in the degradation of proteins, and affected membrane integrity (Supplementary Fig. S3C and Table S4). This agrees with the involvement of membrane damage in increased NCPP development in detached citrus fruit that require extra energy demands to sustain respiration^5,6^. The GO and MapMan analyses also showed that ATP and Gly induced the biosynthesis of nucleotides (Table 2; Fig. 3A,B), which may function as energy sources in plants^19,20^, while DeOGlc + IAc had the opposite effect (Fig. 4). It was noteworthy that both Gly and ATP up-regulated the genes encoding the pyridoxal-5\′-phosphate-dependent enzyme (PRPP) and haloacid dehalogenase-like hydrolase (HAD), which were repressed by DeOGlc + IAc (Supplementary Table S3). PRPP is an essential participant in salvage pathways for ATP regeneration^21^; and the HAD superfamily may increase inorganic phosphate (Pi) for pyruvate and ATP production^22^. These results agree with the 2-fold reduction in ATP decline induced by Gly after fruit detachment (Fig. 1).

Other energy conservation-related processes were differentially expressed only by ATP (Patterns 3 and 6 in Table 2). It was not surprising that ATP negatively modulated glycogen and starch biosynthetic processes (Fig. 4), and BP involved in Suc metabolism (Table 2), which matches the carbon economy and imbalance between carbohydrate demand and import in plants lacking carbohydrate supply^23^. ATP also repressed the BP involved in cell wall catabolism, which agrees with carbon starvation leading to the release of monosaccharides from the cell wall, which are substrates for maintaining ATP production^24^.

There is compelling evidence that carbon availability and energy status are important for the biosynthesis of the aromatic secondary metabolites participating in plant defense via the shikimate pathway^24^. Our results confirmed this because DeOGlc + IAc repressed the BP related to the formation of both the carbon skeletons required for the synthesis of amino acids and the subsequent secondary metabolites involved in cell wall reinforcement or healing in flavedo. Thus DeOGlc + IAc repressed the BP involved in the synthesis of suberin or lignin, which is related to NCPP reduction^6,25^. Once again, these BP were contrarily regulated by both Gly and ATP (Table 2, pattern 1), which reduced NCPP. DeOGlc + IAc brought about a great repression of phenylpropanoid-related genes, like those encoding O-methyl transferases and glutathione S-transferases (GSTs), which were repressed ~30-fold (Supplementary Table S3). These genes are related to cell wall reinforcement, oxidative stress detoxification and NCPP reduction^6,26^. Altogether, our results reinforce that DeOGlc + IAc drives a metabolic readjustment that leads to starvation-induced cell degradation and a reduction in plant defense secondary metabolites, which would favor peel damage in detached citrus fruit.

Our results also support the notion that an altered energy metabolism may involve redox-mediated signaling. ATP and Gly induced the defense responses related to oxidative stress (Supplementary Fig. S3A,B), which agrees with other findings in plants^27^. These responses may contribute to detoxify ROS and to, hence, reduce the NCPP syndrome^6^. Gly had a stronger effect than ATP on glutaredoxin and thioredoxin-encoding genes, and ATP specifically over-represented the superoxide metabolic process and the response to H_2_O_2_ (Pattern 6 in Table 2), which is considered a pivotal signal to enhance stress tolerance in plants^28^. DeOGlc + IAc had a stronger effect on the oxidative stress-induced responses that involve ROS detoxification (including vitamin E and the L-ascorbic metabolic processes) and ROS generation, e.g. the glycolate oxidase complex, which is responsible for H_2_O_2_ generation (Supplementary Table S2). In turn, it repressed the H_2_O_2_ catabolic BP and the genes encoding GSTs, cytochrome P450, alternative oxidase, peroxidase and thioredoxins (Supplementary Table S3, Fig. S3C), which participate in ROS detoxification, and it affected membrane integrity (Supplementary Table S2). These findings indicate that inhibiting energy metabolism may lead to excess ROS, which cannot be detoxified by redox homeostasis to avoid harmful cellular effects. This agrees not only with the DeOGlc + IAc-induced increase noted in peel damage as oxidative stress is a causal factor of NCPP syndrome^6^, but also with the fact that carbon starvation activates ROS production^29^.

The transcriptomic analysis also highlighted the relevance of hormones or related-signaling molecules on the regulation of the metabolic shifts associated with an altered energy metabolism. Gly had a strong effect on the genes with a jasmonate-zim-domain (JAZ/TIFY) (Supplementary Table S3) by participating in JA signaling^30^, and on the BP related to SA (Table 2). The ethylene production of the Gly-treated oranges was higher than that of the control fruit after 3 days (Fig. 7), which agrees with the fact that some Gly-induced processes are regulated by ethylene in citrus fruit^6^. Of them, the BP related to the synthesis of phenylpropanoids and oxylipins, like JA^6^ (Table 2, Supplementary Table S1) merits attention because they participate in limiting cell damage propagation^26,31^. Similarly to Gly, ATP increased ethylene production and induced the genes involved in SA biosynthesis, but barely had any effect on the transcripts involved in the biosynthesis of JA and other oxylipin-derivatives (Table 2, Supplementary Fig. S3B). DeOGlc + IAc also increased ethylene production, but repressed the detection of ethylene stimulus (Table 2). Both energy sources and the inhibitor increased ethylene production, but had an opposite effect on NCPP. Therefore, one suggestion is that ethylene signaling is not relevant for protecting flavedo from NCPP. However, the protective role of this hormone has been demonstrated by applying both ethylene and 1-MCP^5,6^, the latter being a well-known inhibitor of hormone perception in plants. As ethylene negatively regulates its own biosynthesis in citrus fruit, the perception blockage caused by 1-MCP increases hormone production, while augmenting NCPP^6^. Therefore, the apparently contradictory role of ethylene in reducing NCPP when comparing external energy sources and the inhibitor of energy metabolism can be understood by a parallelism between the behavior of DeOGlc + IAc-treated fruit and the effects of 1-MCP on NCPP development. Further support for this idea lies in the fact that DeOGlc + IAc down-regulated genes, like those related to phenylpropanoids metabolism (PAL, OMTs or GSTs), which are up-regulated by ethylene in citrus fruit^6,25^. Moreover, no GO term was commonly regulated by Gly, ATP and DeOGlc + IAc despite the three treatments increasing ethylene production (Table 2, Supplementary Table S1 and Fig. 7). Lastly, the induction of the oligosaccharide biosynthetic BP by ATP (Table 2, pattern 6) is noteworthy because oligosaccharides reduce NCPP in citrus fruit^32^ and favor the synthesis of secondary metabolites with antioxidant activity^33^.

In conclusion, our present findings unravel many effects of Gly and ATP by cushioning the impact of carbon shortage and energy stress on detached citrus fruit. Both energy sources induce common, but also specific, mechanisms grouped in metabolic shifts for the energy conservation and generation of secondary metabolites in order to limit cell damage. Such responses may be mediated by redox and hormone signaling, in which ethylene may play a primary role. JA can also mediate responses induced by Gly, while SA and oligosaccharides might be more important in ATP-induced defense responses. Inhibition of energy metabolism led to oxidative stress- and membrane degradation-related responses. It also repressed JA and SA-related signaling, the detection of ethylene stimulus, and the mechanisms involved in the synthesis of secondary metabolites and cell damage reduction.

## Material and Methods

### Fruit material

Full mature Navelina and Navelate (*Citrus sinensis* (L.) Osbeck) sweet oranges were harvested from adult trees grown in commercial orchards in Liria (Valencia, Spain). Fruit were immediately delivered to the laboratory and divided into groups after selecting those presenting no visual defects or damage. These groups were treated with chemicals or used as controls. In a first experiment, the Navelina fruit was used to test whether Gly and Suc were able to reduce NCPP. The fruit in these groups were sorted into three replicates of 10 fruit each to estimate NCPP during fruit storage at 20 °C and 90–95% RH. These storage conditions were selected to minimize both temperature and water stresses. In a subsequent experiment, Navelate fruit groups were treated with Gly, Suc, AZ + SHAM and DeOGlc + IAc before being stored at 20 °C and 90–95% RH, and were randomly divided into two subgroups. The first subgroup was used to estimate the NCPP incidence, as explained for the Navelina fruit (3 replicates of 10 fruit each per treatment). The second subgroup comprised three replicates of five fruit per storage period, used for the transcriptomic, ATP and ethylene analyses. For the reasons explained in the Results section, the control fruit and the fruit treated with Gly and DeOGlc + IAc were selected for these analyses. The flavedo samples were collected after separating flavedo discs for the immediate ethylene analysis, periodically taken from the total fruit surface in the second subgroup, homogenized in liquid nitrogen and kept at −80 °C for later determinations. Then an experiment was run to examine the effect of ATP on NCPP, ethylene production and the transcriptome of Navelate orange flavedo. To that end, one group of fruit was treated with ATP and a second group was used as the control. Both groups were divided into two subgroups, which were used to estimate NCPP (subgroup 1, 3 replicates of 10 fruit each) and for further analyses (subgroup 2, 3 replicates of 5 fruit each). All the fruit were also stored at 20 °C and 90–95% RH.

### Chemical treatments

Fruit were dipped for 2 min in aqueous solutions containing 50 mM AZ + 50 mM SHAM, 50 mM DeOGlc + 5 mM IAc, 10 mM Suc or 10 mM Gly, dried at room temperature and stored in the dark at 20 °C and 90–95% RH. A solution of 5 mM ATP was also applied periodically for 2 min every 3 days to ensure high ATP levels throughout the experiment. Then 0.5% ethanol was added whenever necessary to dissolve chemicals. Two different controls were added: one was water containing 0.5% ethanol and the other was water alone because Gly and Suc are soluble in water. As we assessed no differences in the NCPP index between both control conditions, their NCPP mean value is shown in Table 1. All the chemicals came from Sigma-Aldrich (St. Louis, MO, USA).

### Determination of NCPP damage

NCPP is manifested as collapsed surface areas. The incidence of NCPP was estimated by calculating the percentage of fruit showing damage. Moreover, NCPP index was calculated^7^ by using a visual rating scale from 0 (no damage) to 4 (severe damage) (Supplementary Fig. S1). The same fruit was used on several evaluation dates. The results are the means of three replicate samples of 10 fruit each.

### RNA isolation

Total RNA was extracted from the frozen flavedo samples as previously described^15^. The RNA concentration was determined spectrophotometrically in a NanoDrop ND-1000 spectrophotometer (Thermo Scientific, Wilmington, DE, USA). Its integrity was verified by agarose gel electrophoresis^15^. RNA quality was further verified by a Model 2100 Total RNA BioAnalyzer with the Eukaryote Total RNA Nano kit (Agilent Technologies).

### cDNA labeling, microarray hybridization, data acquisition and analysis

The transcriptomic changes occurring in the flavedo of Navelate oranges treated with Gly, DeOGlc + IAc or ATP, and stored at 20 °C and 90–95% RH, were determined. Three biological replicate samples per treatment were analyzed by a custom-made citrus cDNA microarray. This microarray contains 44000 unigenes, covers the whole *Citrus sinensis* (L. Osbeck) genome (www.citruseq.org), and was developed using the Agilent platform. In the first experiment, the samples of FH and the fruit treated with Gly and DeOGlc + IAc, and their respective controls, were compared. In the second experiment, the samples from the control and ATP-treated samples were compared. The processing of the RNA samples for the microarray hybridizations was performed according to manufacturer’s two-color protocol (Two-Color Microarray-Based Gene Expression Analysis v. 6.5, Agilent Technologies). A UV-VIS spectrophotometer (Nanodrop 1000, Agilent Technologies, Wilmington, DE, USA) was used to determine dye incorporation; the hybridized microarrays were scanned by a DNA Microarray Scanner (Model G2505C, Agilent Technologies). The microarray data were feature-extracted with Agilent’s Feature Extraction Software (v. 10.7) using the default variables. The same software package was employed to flag the outlier features in arrays. The data analysis, preprocessing and differential expression analysis were performed using the Bioconductor package (under R environment) and the Limma package, respectively. The normexp background correction algorithm was used for the background correction of raw feature intensities. Within-array normalization was done by spatial and intensity-dependent loess. Aquantile normalization was used for normalization among arrays. A gene was considered differentially expressed when it displayed a BH-adjusted p-value < 0.05. DEGs were analyzed by the GO using a hypergeometric analysis (package GOStats)^34^. Thus DEGs were grouped into BP, MF and CC significantly under- or over-represented *versus* the reference sample. In this analysis, only the DEGs showing at least a 2-fold change in expression were considered. A Fisher two-tailed test (p-value < 0.05) was independently performed for the GO analysis of the induced and repressed genes. The Bioconductor package^35^ was used for the PCA analysis. The GeneMaths XT (Applied Maths; http://www.applied-maths.com) software package was used for Heatmap and hierarchical cluster visualization. Finally, the transcriptomic data were loaded into MapMan^36^ (http://www.gabipd.de/projects/MapMan/) to visualize the gene expression data on a metabolic map.

### ATP analysis

ATP was measured in flavedo as previously described^37^ with slight modifications. Briefly, 100 mg of freeze-ground flavedo were extracted with 1.2 mL of chilled 5% trichloro acetic acid (TCA) in a Mini Beadbeater 8 Cell Disruptor (Biospec Products, Inc.) and the extract was centrifuged at 14000 × *g* and 4 °C for 6 min. A 0.6-mL aliquot was taken from the supernatant and the pellet was extracted again with 1.2 mL of chilled 5% TCA. After a 6-minute centrifugation at 14000 × *g* and 4 °C, a 0.6-mL aliquot of the supernatant was taken and combined with the first one. ATP content was estimated after neutralizing and diluting the combined supernatants with 0.1 M Tris acetate buffer (pH 7.75). Five µL of the combined supernatant were added to 4.995 mL Tris acetate buffer to lower the final TCA concentration to below 0.1%. The ATP concentration was determined in the diluted extract by a luciferin/luciferase commercial kit (Sigma, Catalog No. FL-AA, Sigma-Aldrich, St. Louis, MO, USA) by following the manufacturer’s instructions. Two independent diluted extract aliquots per biological replicate sample were analyzed.

### Determination of flavedo ethylene production

Ethylene production from flavedo discs was periodically determined by gas chromatography as previously described^38^. Three replicate samples of 10 flavedo discs each (2 discs from the 5 fruit selected for the transcriptomic analysis in each replicate) were used.

### Statistical analysis

A mean comparison (Tukey’s test) was made to test whether the mean values of NCPP, ethylene production and ATP concentration significantly differed (P ≤ 0.05) with the Statgraphics Plus 4.0 Software (Manugistics, Inc.). Values are given as the mean of three biological replicate samples.

## Supplementary information

Supplementary information Supplementary Table 2 Supplementary Table 3

## Data Availability

All the data generated or analyzed in this study are included in this published article (and its Supplementary Information files).
