# Supplementary material for: Insights into the regulation of molecular mechanisms involved in energy shortage in detached citrus fruit
Source: Sci Rep. 2020 Jan 24;10:1109. doi: 10.1038/s41598-019-57012-7 (PMC6981168; doi:10.1038/s41598-019-57012-7)

Insights into the Regulation of Molecular Mechanisms Involved in Energy Shortage in Detached Citrus Fruits

Paco Romero, Fernando Alférez, Beatriz Establés-Ortiz, and María T. Lafuente

Supplementary Information: Supplementary TableS1 and Supplementary Figures S1-S4

Table S1. Gene ontology (GO) analysis (GOStats, $P < 0.05$) of molecular functions (MF) and cellular components (CC) over- (↑) or infra-represented (↓) in the flavedo of the Gly-, DeOGlc+IAc- and the ATP-treated fruits, with respect to their controls. Processes were grouped according to their regulation pattern considering the ability of the treatments to provide energy or to interfere with energy metabolism. Three biological replicates from each condition were used.

GO Category	GO Code	GO Term	Gly	ATP	DeOGlc+IAc
**Pattern 1: Commonly regulated by Gly and ATP, and contrary to DeOGlc+IAc**					
MF	GO:0047763	caffeate O-methyltransferase activity	↑	↑	↓
**Pattern 2: Contrarily regulated between Gly and DeOGlc+IAc**					
MF	GO:0080031	methyl salicylate esterase activity	↑		↓
MF	GO:0080032	methyl jasmonate esterase activity	↑		↓
MF	GO:0080030	methyl indole-3-acetate esterase activity	↑		↓
**Pattern 3: Contrarily regulated by ATP and DeOGlc+IAc**					
MF	GO:0009815	1-aminocyclopropane-1-carboxylate oxidase activity		↑	↓
MF	GO:0031418	L-ascorbic acid binding		↑	↓
MF	GO:0008757	S-adenosylmethionine-dependent methyltransferase activity		↑	↓
MF	GO:0005506	iron ion binding		↑	↓
MF	GO:0030170	pyridoxal phosphate binding		↑	↓
MF	GO:0016836	hydro-lyase activity		↑	↓
MF	GO:0045330	aspartyl esterase activity		↑	↓
MF	GO:0016702	oxidoreductase activity, acting on single donors with incorporation of molecular oxygen, incorporation of two atoms of oxygen		↑↓	↓
MF	GO:0045486	naringenin 3-dioxygenase activity		↑	↓↑
MF	GO:0008878	glucose-1-phosphate adenylyltransferase activity		↓	↑
MF	GO:0004565	beta-galactosidase activity		↓	↑
MF	GO:0004568	chitinase activity		↓	↑
MF	GO:0008171	O-methyltransferase activity		↓	↑
CC	GO:0016021	integral to membrane		↓	↓↑
CC	GO:0009341	beta-galactosidase complex		↓	↓↑
**Pattern 4: Commonly regulated by Gly and ATP**					
MF	GO:0070330	aromatase activity	↑	↑	
MF	GO:0016682	oxidoreductase activity, acting on diphenols and related substances as donors, oxygen as acceptor	↑	↑	
**Pattern 5: Regulated by Gly**					
MF	GO:0016740	transferase activity	↓		

**Pattern 6: Regulated by ATP**			
MF	GO:0008378	galactosyltransferase activity	↑
MF	GO:0000213	tRNA-intron endonuclease activity	↑
MF	GO:0004143	diacylglycerol kinase activity	↑
MF	GO:0009672	auxin:hydrogen symporter activity	↑
MF	GO:0005509	calcium ion binding	↑
MF	GO:0042803	protein homodimerization activity	↑
MF	GO:0042803	protein homodimerization activity	↑
CC	GO:0000214	tRNA-intron endonuclease complex	↑
CC	GO:0009523	photosystem II	↑
MF	GO:0004866	endopeptidase inhibitor activity	↓
MF	GO:0016829	lyase activity	↓

Figure S1. Visual rating scale from 0 (no damage) to 4 (severe damage) of non-chilling peel pitting (NCPP) syndrome in Navelate oranges.

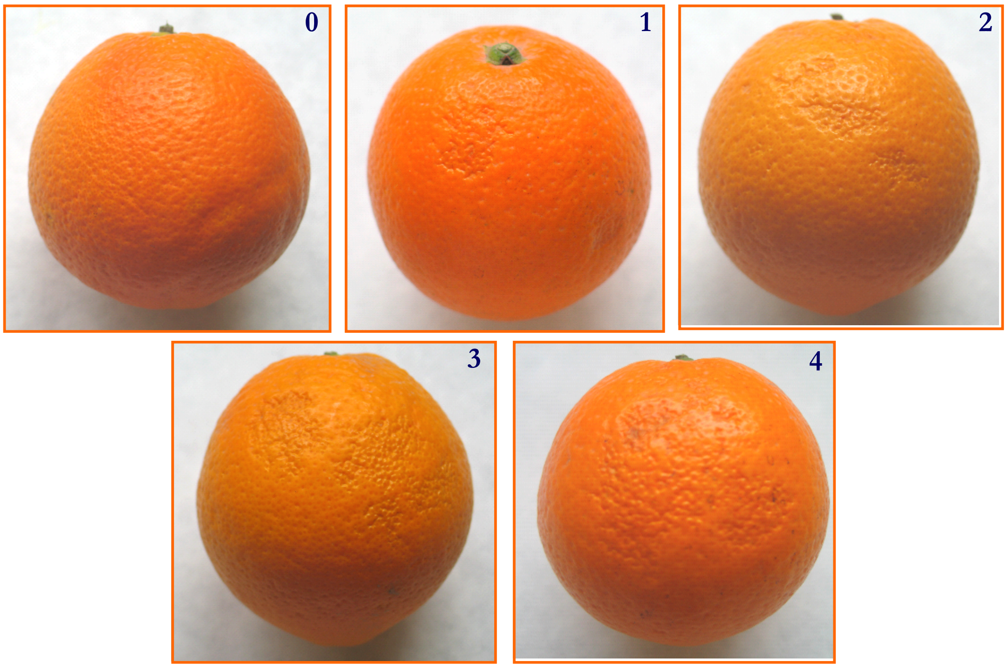

Figure S2. Transcriptomic comparative analyses of the flavedo of Navelate oranges treated with 50 mM DeOGlc+5 mM IAc or with 10 mM Gly. A) Number of DEGs (SAM, FDR<0.01) for the comparisons between fruits treated with DeOGlc+IAc and their control fruits, and between fruits treated with Gly and their control fruits. Only DEGs showing at least a 2-fold change in expression are included. Principal Component Analysis (PCA) (B), and Heatmap and hierarchical cluster analysis (C) based on large-scale transcriptional profiles of the DEGs satisfying an ANOVA test (P<0.05) for all conditions represented: FH: Freshly harvested fruits; DeOGlc+IAc: fruits treated with DeOGlc+IAc and kept for 3 days at 20 ºC and 90-95% RH; C_ DeOGlc+IAc: control fruits of the DeOGlc+IAc-treated sample; Gly: fruits treated with Gly and kept for 7 days at 20 ºC and 90-95% RH; C_Gly: control fruits of the Gly-treated sample. Heatmap colours vary from dark blue to dark red on an intensity scale ranging from 6 to 18. Three biological replicates from each condition were used for the analyses.

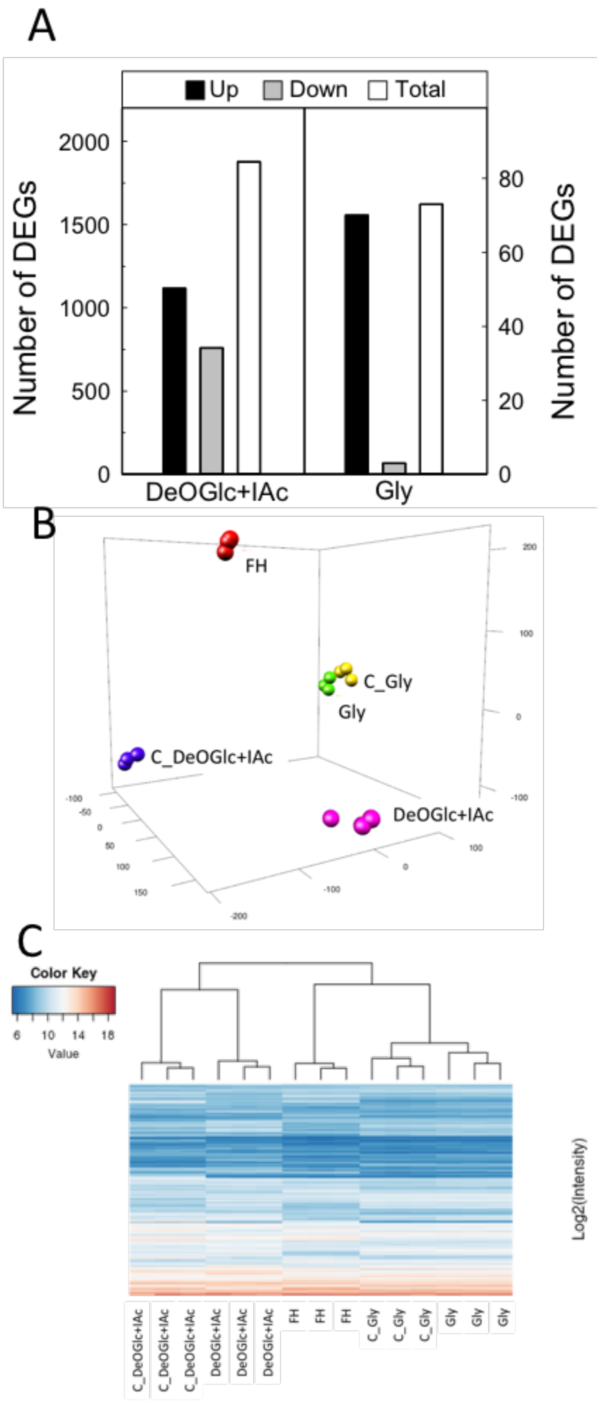

Figure S3. Regulatory overview using MapMan[36] when comparing transcript accumulation in the flavedo of Gly- (A), ATP- (B), and DeOGlc+IAc-treated (C) fruits respect to their control non-treated samples. Fruits were kept under darkness at 20 ºC and 90-95% RH. Red and blue squares represent genes with decreasing and increasing transcript levels in treated fruits, respectively, as compared to control samples. Color scale is indicated in the figure.

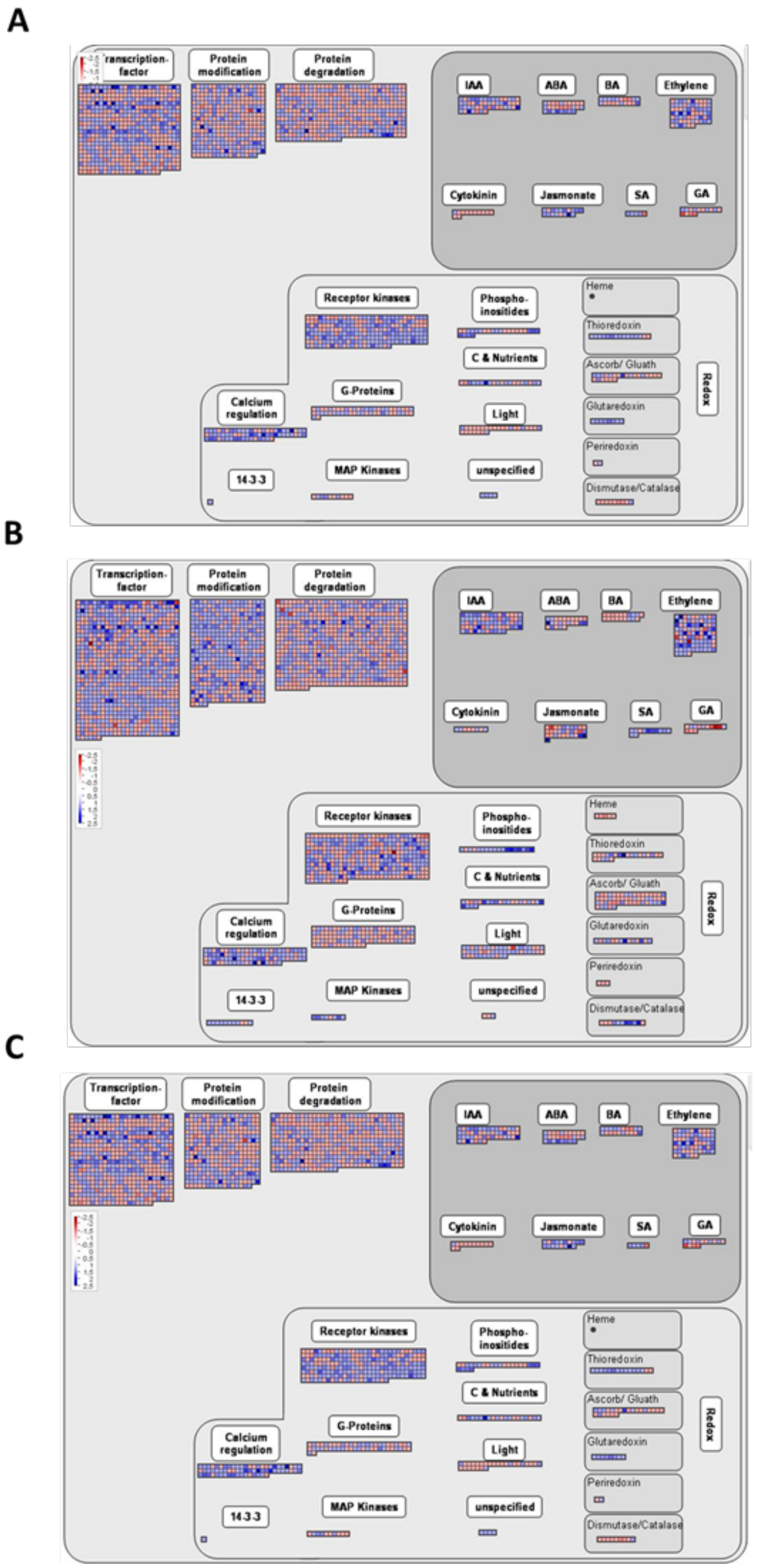

Figure S4. TCA cycle detail using MapMan[36] when comparing transcript accumulation in the flavedo of Gly- (A), ATP- (B), and DeOGlc+IAc-treated (C) fruits respect to their control non-treated samples. Fruits were kept under darkness at 20 ºC and 90-95% RH. Red and blue squares represent genes with decreasing and increasing transcript levels in treated fruits, respectively, as compared to control samples. Color scale is indicated in the figure.

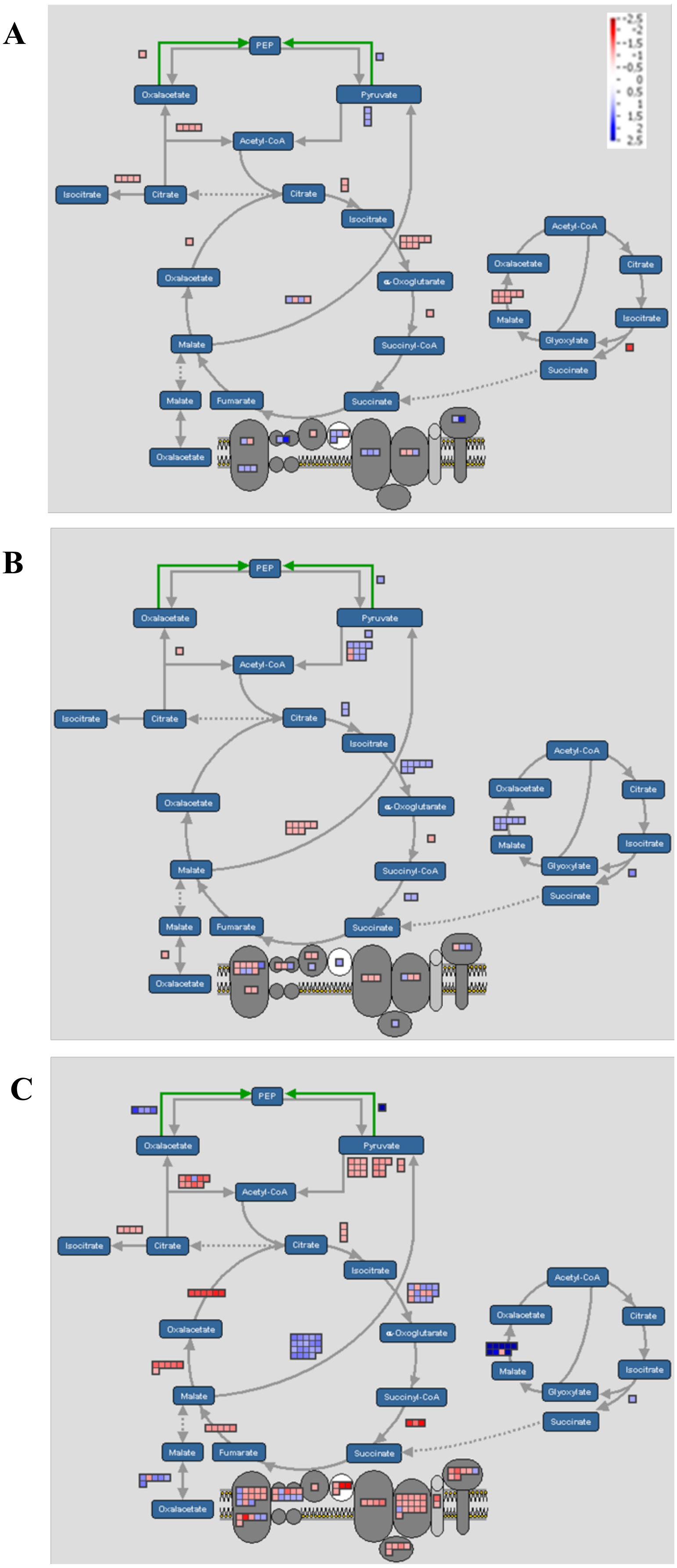

Supplement: Supplementary file 1 — Supplementary information [file 41598_2019_57012_MOESM1_ESM.pdf]
